# Supplementary material for: Stoic: fast and accurate protein stoichiometry prediction
Source: Bioinformatics. 2026 Aug 21;42(Suppl 2):btag448. doi: 10.1093/bioinformatics/btag448 (PMC13501280; doi:10.1093/bioinformatics/btag448)
Supplement: Supplementary file 1 [file btag448_supplementary_data.pdf]

```
StoichiometryModel(
❄(seq_embed_model): Esm2()
  (feature_pooling_strategy): SelfAttentionPooling(
    (layer_norm): InstanceNorm1d(1280, eps=1e-05, momentum=0.1, affine=True, track_running_stats=False)
    (norm): InstanceNorm1d(1280, eps=1e-05, momentum=0.1, affine=True, track_running_stats=False)
    (self_attention): MultiheadAttention(
      (out_proj): NonDynamicallyQuantizableLinear(in_features=1280, out_features=1280, bias=True)
    )
    (linear): Sequential(
      (0): Linear(in_features=1280, out_features=1280, bias=True)
      (1): LayerNorm((1280,), eps=1e-05, elementwise_affine=True)
      (2): GELU(approximate='none')                                   Interface Loss
      (3): Dropout(p=0.2, inplace=False)
      (4): Linear(in_features=1280, out_features=1, bias=True)
    )                                                    Pooling Mechanism: 8207361 params
  )
  (seq_feature_encoder): GCNConv(
    (dropout): Dropout(p=0.2, inplace=False)
    (activation): GELU(approximate='none')
    (gcn): GCN(1280, 1280, num_layers=1)                   Context Encoder: 1639681 params
  )
  (node_classifier): Sequential(
    (0): Linear(in_features=1280, out_features=320, bias=True)
    (1): LayerNorm((320,), eps=1e-05, elementwise_affine=True)
    (2): GELU(approximate='none')
    (3): Dropout(p=0.2, inplace=False)                       Complex Loss
    (4): Linear(in_features=320, out_features=13, bias=True)
  )
)
                                                          Prediction Head: 414733 params
```

Supplementary Figure S1 **Detailed architecture of Stoic.**

Supplementary Table S1 Mapping of prediction classes between Seq2Symm and *Stoic*.

Seq2Symm	Stoic
C1	1
C17	17
C2	2
C3	3
C4	4
C5	5
C6	6
C9	9
D12	24
D2	4
D3	6
D4	8
D5	10
I	60
O	24
T	12,24
CX	undefined
DX	undefined
H	undefined
HOTI	undefined

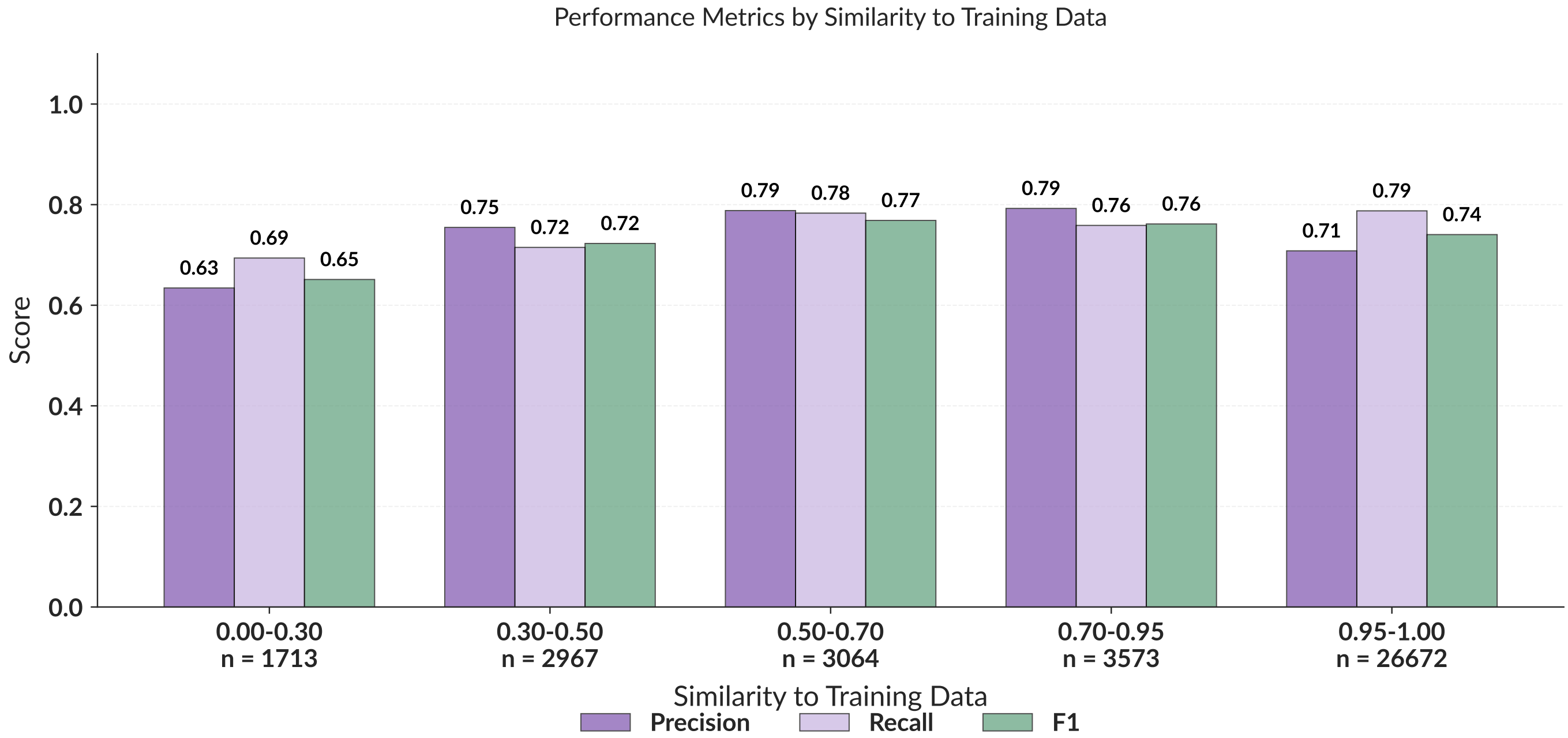

Supplementary Figure S2 Copy number prediction performance of Stoic depending on similarity to the train dataset.

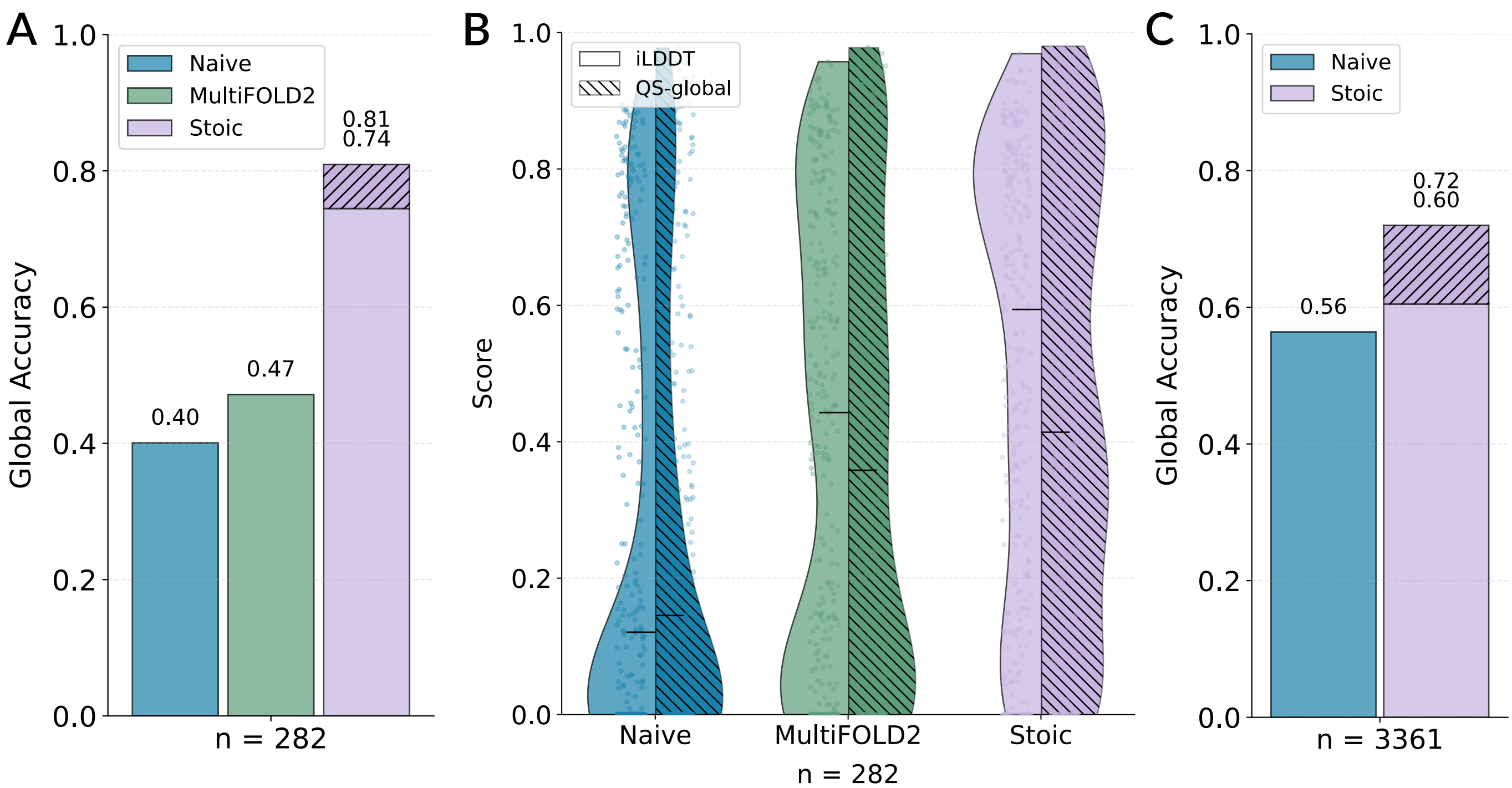

Supplementary Figure S3 Stoichiometry and structure prediction performance. A) Global stoichiometry prediction accuracy on the CAMEO subset for which MultiFOLD2 (green) predictions are available, comparing Naive prediction (copy number of one assigned to every entity) and *Stoic* (purple; best-of-3 shown as a dashed bar). Monomers are excluded because iLDDT and QS-global are not defined for them. **B)** Distribution of iLDDT (solid) and QS-global (dashed) scores for AlphaFold3 models generated using Naive (blue), MultiFOLD2 (green), and *Stoic* (purple) predicted stoichiometries. **C)** Global stoichiometry prediction accuracy on the Complex Portal (Balu et al., 2025) dataset, for which experimental stoichiometry annotations are available.
